# Multireference *Ab Initio* Investigation on Ground and Low-Lying Excited States: Systematic Evaluation of *J*–*J* Mixing in a Eu^3+^ Luminescent Complex

**DOI:** 10.1021/acs.inorgchem.0c02956

**Published:** 2020-12-15

**Authors:** Luca Babetto, Silvia Carlotto, Alice Carlotto, Marzio Rancan, Gregorio Bottaro, Lidia Armelao, Maurizio Casarin

**Affiliations:** † Dipartimento di Scienze Chimiche, Università degli Studi di Padova, via F. Marzolo 1, 35131 Padova, Italy; ‡ Institute of Condensed Matter Chemistry and Technologies for Energy (ICMATE), National Research Council (CNR), c/o Department of Chemistry, University of Padova, via F. Marzolo 1, 35131 Padova, Italy

## Abstract

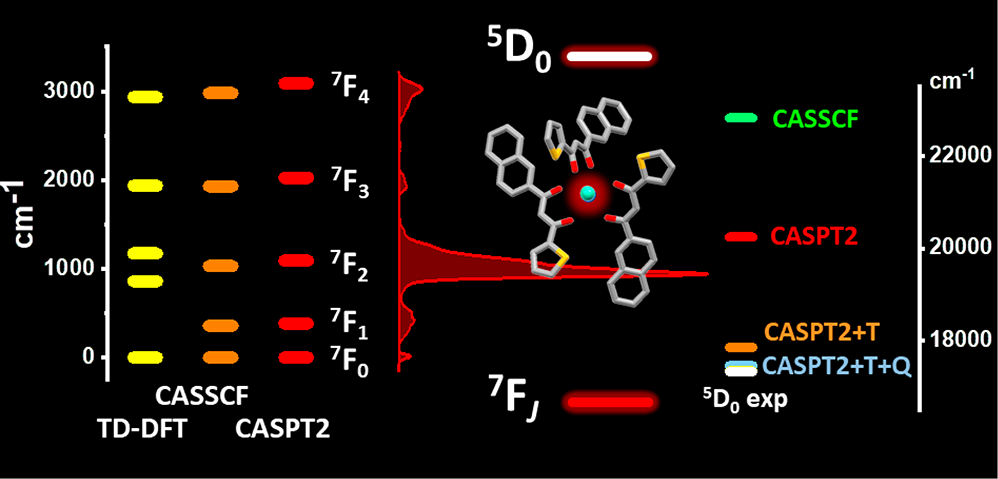

A theoretical protocol combining density functional theory (DFT) and multireference (CAS) calculations is proposed for a Eu^3+^ complex. In the complex, electronic levels of the central Eu^3+^ ion are correctly calculated at the CASPT2 level of theory, and the effect of introducing different numbers of states in the configuration interaction matrices is highlighted as well as the shortcomings of DFT methods in the treatment of systems with high spin multiplicity and strong spin–orbit coupling effects. For the ^5^D_0_ state energy calculation, the inclusion of states with different multiplicity and the number of states considered for each multiplicity are crucial parameters, even if their relative weight is different. Indeed, the addition of triplet and singlets is important, while the number of states is relevant only for the quintets. The herein proposed protocol enables a rigorous, full *ab initio* treatment of Eu^3+^ complex, which can be easily extended to other Ln^3+^ ions.

## Abstract 2

A theoretical protocol combining density functional theory and multireference calculations is proposed for a Eu^3+^ complex. For the ^5^D_0_ state energy calculation, the inclusion of states with different multiplicity and the number of states considered for each multiplicity are crucial parameters. The herein proposed protocol enables a rigorous, full *ab initio* treatment of Eu^3+^ complex, which can be easily extended to other Ln^3+^ ions.

## 1 Introduction

In recent decades, lanthanides have been employed in a wide variety of applications spreading from energy production to life sciences.^1^ They are a fundamental element in light-emitting diodes, displays, lasers, telecommunications, sensors, molecular thermometers, lighting systems, and biological immunoassays and imaging.^2−4^ Among lanthanides, the Eu^3+^ ion has had an increasingly relevant role as a luminescent activator in different classes of materials due to its high efficiency as a red light emitter.^5^ Moreover, its energy level structure is relatively simple, and the ground (^7^F_0_) and the emitting (^5^D_0_) states are not degenerate; hence, it is possible to monitor Eu^3+^ emission and excitation transitions also in a host lattice.^6,7^ Some ^5^D_0_ → ^7^F*_J_* electronic transitions are very sensitive to the local environment surrounding the ion; therefore, Eu^3+^ can be used as a spectroscopic probe for investigating structural properties of the material in which it is embedded.^8,9^ This characteristic results in the extensive use of this ion to determine the local symmetry of an ion site,^10,11^ to test the crystal defects, to evaluate the crystal field strength,^12^ and to rationalize the thermal treatment effects on oxides.^13^ Literature highlights the importance of accurate determination of the electronic states of the Eu^3+^; hence, the development of new methods and the nonstandard application of the existent theoretical tools to correctly include the not always negligible effects of the ligand field on 4f states are the new frontier in the *ab initio* treatment of this ion.

Theoretical studies on Eu^3+^ complexes consist of two main approaches: (i) semiempirical methods often parametrized for a single class of compounds (e.g., the LUMPAC^14,15^ program) and (ii) density functional theory (DFT) and multireference *ab initio* methods.^16−18^ Only the latter approaches allow in principle to tackle a wide range of systems, but there is not a general consensus on how to carry out these high-level calculations on molecular systems, especially when multireference methods such as complete active space self-consistent field (CASSCF) and complete active space second-order perturbation theory (CASPT2) are considered.^17,19−23^

Some work has been done in investigating the effect of including different electronic states on the energy of low-lying excited states in isolated Eu^3+^ ion, but without a thorough and systematic procedure and neglecting the effects of the surrounding environment.^24^ CASSCF/CASPT2 methods have been also applied to disordered systems, such as Eu^3+^-doped glasses.^25−27^ In these works, the environment is treated implicitly through the use of a model potential.

In the case of molecular systems, for which the chemical environment needs to be treated explicitly, there is still uncertainty on where to focus the attention: some authors evaluate the influence of excited states with different spin but neglect the effects of second-order perturbations;^17^ others recognize the importance of dynamic correlation and employ a reasoned number of quintet states, but they do not include states with different spin multiplicity such as triplets and singlets.^19^ As a whole, in most cases in the literature it can be seen that the energy of the ^5^D_0_ emitter is not correctly reproduced.^17,19,20,22,23,28^

Recently, hybrid approaches combining the computationally efficient qualities of semiempirical methods and the accuracy of full *ab initio* calculations—the CERES^29^ program is one prime example—have started to catch on. In these suites of programs, a specific *ab initio* protocol is optimized and set up for the determination of certain observables. The CERES program, for instance, focuses on calculations of magnetic properties of lanthanide complexes, also limited to Eu^3+^, in an efficient way by employing some approximations in the description of the electronic states, which are perfectly valid if we limit the attention to the magnetic properties. In particular, magnetic properties are not significantly influenced by higher energy excited states and are mostly attributed to the ground state (GS) manifold. The program therefore does not include second-order perturbations (CASPT2), which are only relevant when excited states are considered.

**Figure 1:**
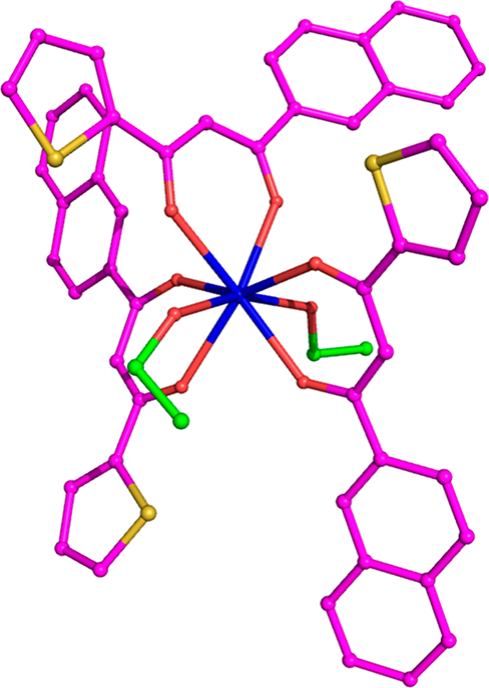
A ball and stick representation of the Eu complex with the antenna ligands (three) in magenta and the ancillary ligands (two) in green. The coordination number of the Eu^3+^ ion (the central blue sphere) amounts to 8. Magenta, red, yellow, and blue spheres are C, O, S, and Eu atoms, respectively.

Literature^18,30,31^ demonstrated that when considering excited states, dynamic correlation in the form of second-order perturbation theory needs to be introduced, but the role of the mixing and the choice of the relevant states is still under discussion. The main aim of this contribution is to present a general theoretical protocol based on a combination of DFT and multireference methods to gain detailed information about electronic states of the Eu^3+^ ion, with the possibility to extend the results to other lanthanides. The protocol has been validated for a Eu^3+^ complex, general formula EuL_3_(EtOH)_2_, where L is a β-diketone (see Figure 1).

The main aim of the study is the calculation of the excited (^5^D_0_) and ground (^7^F*_J_*) energy levels for Eu complex. The role played by the number of excited states adopted in the multireference calculations and the relevance of the mixing of states with the same *J* value will be rationalized for different computational approaches. Moreover, the absorption spectrum will be simulated to understand how the electronic properties of the complex depend on the Eu^3+^ and ligand fragments. Because of the relatively simple electronic structure where the ground (^7^F_0_) and the emitting (^5^D_0_) states are not degenerate, Eu^3+^ will be then herein considered as a case study to showcase the effect of including different states, with the awareness that the obtained results will have a general validity and could be straightforwardly transferred to whatever Ln^3+^ ion.

## 2 Methods

### Experimental Details

The studied complex has the general formula EuL_3_(EtOH)_2_, where L is a β-diketone which features a thienyl and a naphthyl group as substituents. The ligand and [EuL_3_(EtOH)_2_] compounds were prepared as previously reported.^32^ Absorption spectra were recorded on a CARY5000 double-beam spectrophotometer in the 300–800 nm range, with a spectral bandwidth of 1 nm. The contribution due to the toluene solvent was subtracted. Photoluminescence spectrum was acquired with a Horiba Fluorolog 3-22 spectrofluorometer.

### Computational Details

DFT calculations have been performed by using the Amsterdam Density Functional (ADF) package (ver. 2013.01),^33−35^ while multireference *ab initio* calculations have been run by exploiting the OpenMolcas package.^36−38^

The generalized gradient approximation (GGA) PBE^39−42^ functional coupled to a TZ2P basis set has been employed to optimize the Eu complex geometry. Core–shells up to level 4d for Eu, 2p for P and S, and 1s for O and C have been kept frozen throughout the calculations. Scalar relativistic effects have been included by adopting a two-component Hamiltonian with the zeroth-order regular approximation (ZORA).^43−45^ Once again, frequency calculations have been performed to ensure the geometry optimization had reached a minimum in the potential energy hypersurface. The complex absorption spectrum has been simulated at the same level of theory of the free ligands by using the statistical average of orbital potential (SAOP) with a TZ2P basis set, as the transitions are ligand-centered in nature (see the Results and Discussion section).

Complete active space self-consistent field (CASSCF) calculations have been performed on a model system that maintains the same coordination sphere as the full complex at the DFT optimized geometry (see details in the discussion) by using the all-electron Gaussian-type atomic natural orbital-relativistic core-correlated basis set contracted to TZP quality (ANO-RCC-VTZP).^46,47^ Scalar relativistic effects have been included by means of the two-component second-order Douglas–Kroll–Hess (DKH) Hamiltonian in its scalar form.^48^ Spin–orbit coupling (SOC) has been treated by state interaction between the CASSCF wave functions by using the restricted active space state interaction (RASSI) program.^49^ The SOC operator matrix has been calculated from the atomic mean-field (AMFI) approximation,^50^ while dynamic correlation has been included by using the complete active space second-order perturbation theory (CASPT2) method.^51,52^ The active space has been selected by including six electrons in the seven 4f orbitals, equating to a CAS(6,7) calculation. A multitude of states for each spin multiplicity have been evaluated, and further details are reported in the Results and Discussion section. As far as the correlation orbital space for the CASPT2 calculation is concerned, it has been limited to the central Eu^3+^ ion and the ligand donor atoms (*AFREeze* keyword). Just for comparison, the Eu^3+^ emitter state ^5^D_0_ has been also calculated by considering the lowest energy spin-flip^53,54^ TD-DFT/LB94^55^ transition between the GS characterized by six unpaired electrons and a state with four unpaired electrons; the ^7^F*_J_* states energies have also been evaluated at the TD-DFT/LB94 level of theory.

The specific influence of the solvent effects and of the dispersion corrections on this ligand was investigated in detail in a previous study.^56^ The negligible variations with respect to the gas phase calculations for toluene allows us to avoid including the solvent in the calculations.

## 3 Results and Discussion

### GS Geometry

**Figure 2:**
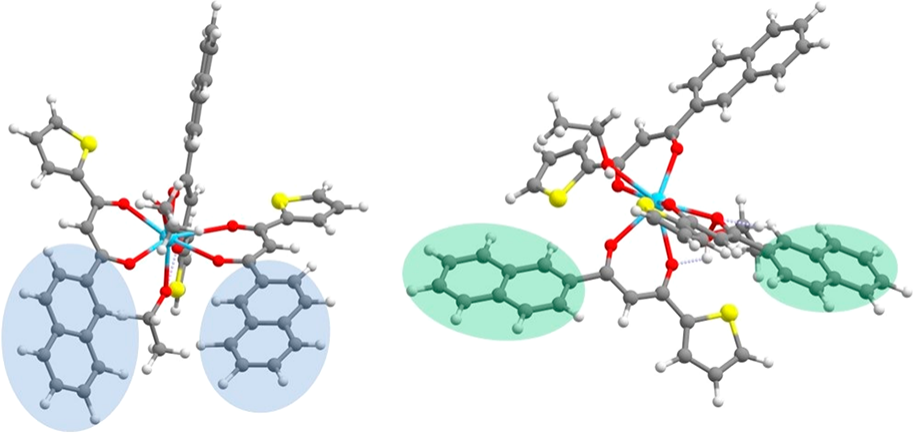
*Cis* (left) and *trans* (right) isomers of the Eu complex. Gray, white, red, yellow, and blue spheres are C, H, O, S, and Eu atoms, respectively.

The crystal structure of the Eu complex is not available, which makes DFT calculations the only source of information about structural properties. As such, the accuracy of DFT has been recently tested on similar Eu complexes characterized by the presence of two thienyl groups substituents,^32^ where the PBE XC functional coupled to a TZ2P basis set accurately reproduced the crystal structure geometry. The same level of theory has been then herein used to optimize the Eu complex. The ligand symmetry implies that the complex may assume *cis* and *trans* configurations depicted in Figure 2 and defined as follows: in the former, the polyaromatic hydrocarbon moieties of the two almost coplanar ligands are on the same side, while in the latter they are opposed.

To obtain the optimized geometries of both stereoisomers, we started from *cis* or *trans* configurations. Independently from the starting configuration, the final geometry converged toward the *trans* one, probably due to the significant steric hindrance between the aromatic fragments in the *cis* form. The impossibility to achieve the *cis* form suggests that this form is not stable enough to provide any contribution to experimental measurements.

### Absorption Spectra

**Figure 3:**
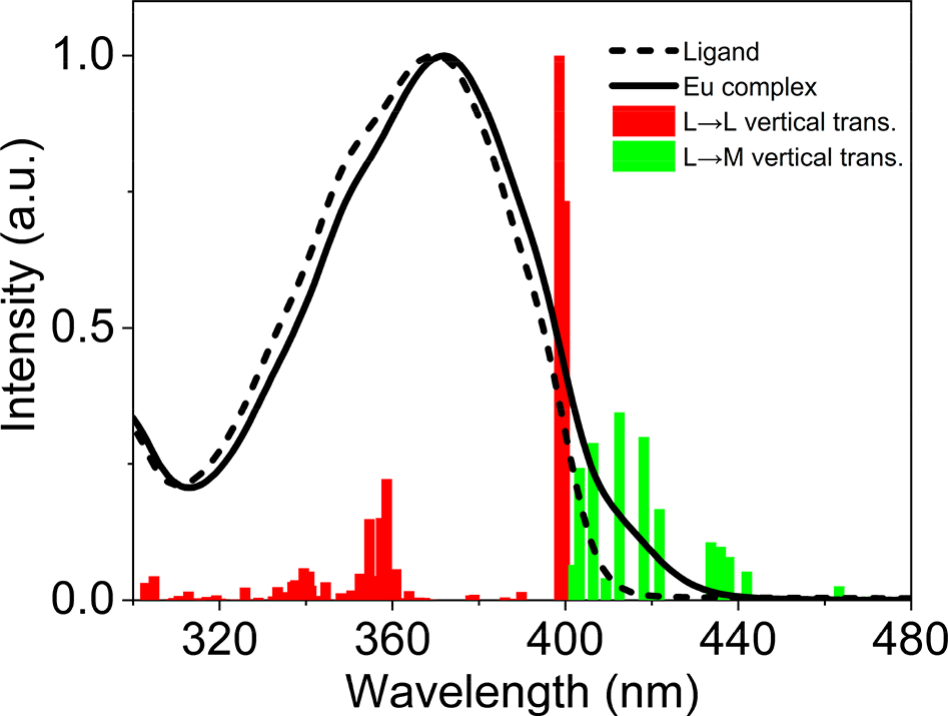
Calculated SAOP vertical transitions (bars) for the Eu complex. The red and the green bars represent the ligand centered and the ligand-to-metal-charge-transfer vertical transitions, respectively. The experimental spectra for the Eu complex (solid line) and the isolated ligand (dashed line) are reported. Ligand and complex absorption spectra are obtained in toluene with a concentration of 5 × 10^–6^ and 3 × 10^–6^ M, respectively.

To understand the role of the ligand and the Eu^3+^ ion on the electronic properties and to follow the variation from the isolated fragments to the complex, the absorption spectra of isolated ligands and the Eu complex are compared. Figure 3 reports the overlap between the ligand and Eu complex absorption spectra. Even though similar, the two UV–vis spectral patterns are not identical. Such evidence suggests that light absorption in the complex is almost completely localized on the ligand, and a detailed analysis of the ligand absorption spectra and the role of the vibronic progression is reported in our previous investigation.^56^

The main difference in the two experimental spectra is a weak but clearly visible shoulder at ∼420 nm, which is missing from the ligand pattern (Figure 3). The efforts are then focused on elucidating the nature of this mismatch; as such SAOP vertical transitions have been calculated for the Eu complex (colored bars in the Figure 3). Unsurprisingly, the UV–vis spectrum of the complex is dominated by ligand-based transitions of the same nature as that of the free ligand (red bars in Figure 3), as highlighted by molecular orbital analysis (see Table S1 in the Supporting Information). A direct comparison between the isolated ligand and the Eu complex main transitions further highlights the similarity of the initial and final molecular orbitals (see Figure S1) and that the complex spectrum is only weakly affected by the presence of the central Eu^3+^. Other than that, there are several weak transitions lying at lower energies (∼420 nm) with a ligand-to-metal-charge-transfer (LMCT) character (green bars in the Figure 3). The weak shoulder characterizing the complex spectra can therefore confidently be assigned to LMCT transitions (see Table S1). These results confirm that the ligand maintains the electronic properties of the isolated condition; hence, the ligand and the metal center can be considered practically independent.^56^ Even if independent, these two fragments can interact, and new properties arise from this interaction, such as the shoulder in the complex spectrum due to the LMCT transitions. A clear trace of this interaction is also observed in the variation of the Eu^3+^ ground state (^7^F*_J_*) energies going from the Eu^3+^ isolated ion to the Eu complex.

### TD-DFT Calculations for ^5^D_0_ and ^7^F_*J*_ Levels

As for the Eu^3+^-centered transitions, it has to be kept in mind that DFT, a *single-determinant* method, is not well suited to investigate the Ln^3+^ electronic properties, and the adopted software package (ADF) does not currently allow for a *self-consistent* treatment of spin–orbit coupling in open-shell systems, which is the leading perturbation term for rare earths after electron repulsion. Furthermore, conventional TD-DFT cannot calculate transitions between terms with different spin multiplicities in open-shell systems; a variation of the method called *spin-flip* TD-DFT is required, in which electrons initially located in α orbitals are only excited to β orbitals, and vice versa. At a first glance, TD-DFT transitions calculated by exploiting the LB94 functional in which only scalar relativistic effects have been included seem to be in good agreement with experimental evidence (Table 1).

**Table 1:** ^7^F*_J_* and ^5^D_0_ State Energies (in cm^–1^) Calculated at the Scalar Relativistic TD-DFT/LB94 and the RASSI-CAS(6,7)PT2 Level for the Eu^3+^ Model Complex^a^

	TD-DFT	CAS(6,7)	CAS(6,7)PT2	Eu complex exp.	Eu^3*+*^ free ion exp.^5^
Ground State					
^7^F_0_	0	0	0	0	0
^7^F_1_	860	359	384	392	379
^7^F_2_	1171	1029	1091	1119	1043
^7^F_3_	1935	1929	2025	1955	1896
^7^F_4_	2934	2977	3088	2898	2869
^7^F_5_	3995	4110	4214	//	3912
^7^F_6_	6440	5294	5370	//	4992
Excited State					
^5^D_0_	16339	22789	20214	17302	17227

a Each ^7^F*_J_* term for CASSCF calculations is taken as the barycenter of the respective manifold generated by crystal field splitting.

Table 1 reports ^7^F*_J_* and ^5^D_0_ state energies for both Eu^3+^ free ion and Eu complex to demonstrate that the variation between them is small but not negligible. The coordination environment influences the Eu^3+^ energy levels, and this effect has to be considered.

**Figure 4:**
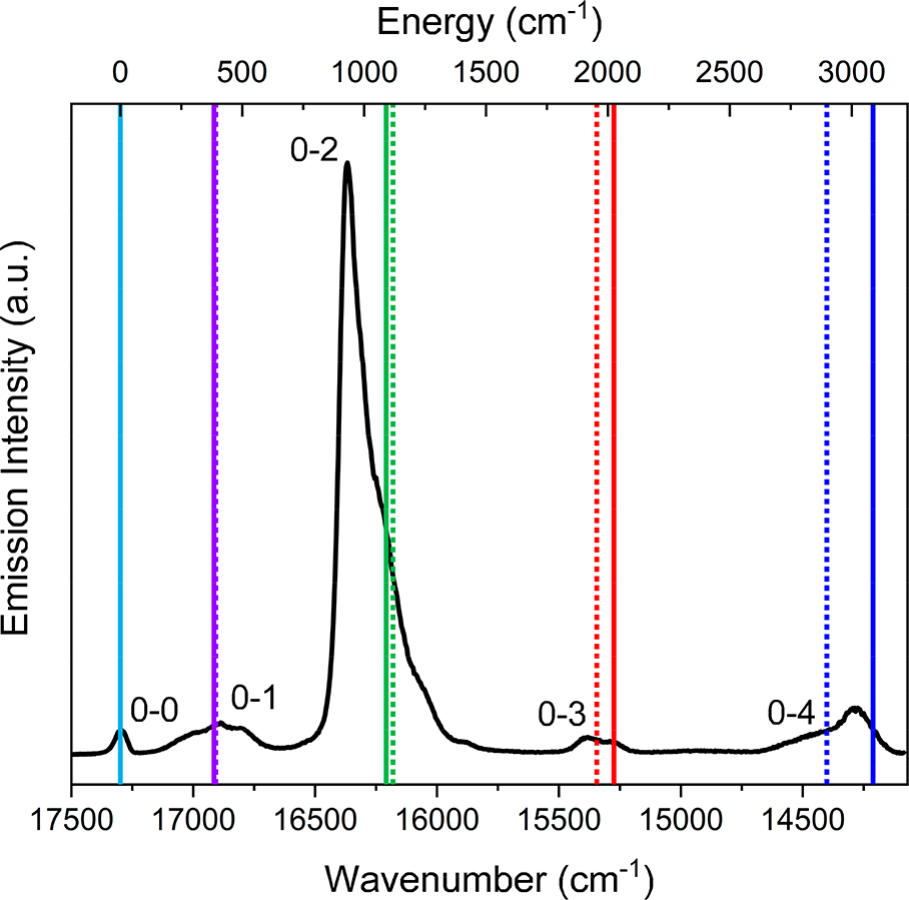
The solid black line is the experimental emission spectrum of the Eu complex. The solid and dotted lines are the ^7^F*_J_* CAS(6,7)PT2 and experimental values (see Table 1), respectively. Details for the experimental values are reported in the text.

To allow a direct and reliable comparison between experimental and calculated data, the experimental energy of the different ^7^F*_J_* manifolds are obtained as arithmetic mean of the initial and final energy of each ^5^D_0_ → ^7^F*_J_* multiplets (*J* = 0, 1, 2, 3, and 4, Figure 4, dotted lines), deduced from the emission spectrum (Figure 4, solid black line). The calculation of the average wavenumber of the transitions using the intensity of experimental spectrum as weight factor^5^ is not a good choice in our case because the calculated values cannot be correlated to any oscillator strength and hence cannot be weighted.

When looking at the first column of Table 1, it must be remembered that experimental lines arise from transitions between the different ^7^F*_J_* states generated by the SOC interaction, which is not taken into account in TD-DFT calculations^57^ and TD-DFT calculations performed for the Eu complex are only purely 4f*–*4f in nature. To appropriately describe the electronic states of the Eu^3+^ ion, higher level calculations are therefore necessary.

### Multireference Calculations for ^5^D_0_ and ^7^F_*J*_ Levels

**Figure 5:**
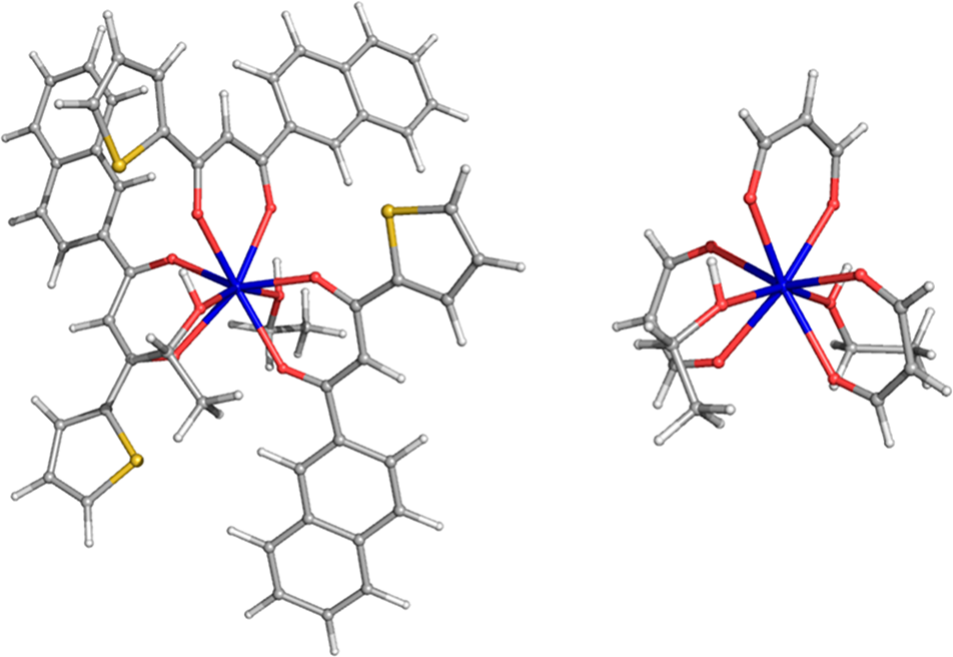
Eu complex (left) and its simplified model (right) obtained by substituting the ligand with malondialdehyde. Gray, red, yellow, white, and blue spheres are C, O, S, H, and Eu atoms, respectively.

The valence electrons for Eu^3+^ ions reside in orbitals which are shielded from the environment by the closed 5s^2^ and 5p^6^ outer shells: the intensity of crystal field effects, which lift the degeneracy of the electronic terms originated from the 4f^*n*^ configuration, is then greatly mitigated by comparison with transition metal ion complexes. Moreover, SOC scales with the fourth power of the atomic number *Z*, thus overwhelming, in heavy elements such as lanthanides, effects associated with the crystal field splitting. Eu^3+^-based transitions are therefore expected to be almost in the same energy range even for a significantly different environment, as widely confirmed by the literature.^58−60^ All of this allows to carry out multireference calculations by focusing on the Eu^3+^ center and modeling the antenna ligands in a simplified fashion, that is, by maintaining the actual complex coordination sphere with the antenna ligands only featuring the fragment directly coordinated to the Eu^3+^ ion. The Eu complex has been then modeled by substituting the ligand with a much simpler one, but with a similar structure (malondialdehyde) to preserve the Eu^3+^ coordination sphere geometry (Figure 5). The positions for the atoms that are taken from the full complex are kept fixed, while the hydrogen atoms replacing the aromatic fragments have been reoptimized at the same level of theory.

The *static* correlation, arising from the multideterminant nature of the wave function, has been recovered via spin-adapted state-averaged CASSCF followed by state interaction with spin–orbit coupling. Such a procedure, able to properly describe the GS manifold, yields a series of electronic states linkable to Russell–Saunders terms. Besides static correlation, the evaluation of the excited state energies needs the inclusion of *dynamic* correlation as well in the form of second-order perturbation theory on the CASSCF wave function (CASPT2). As such, it is necessary to define two parameters in CASSCF/CASPT2 calculations: (i) the active space and (ii) the dimensions of the configuration interaction (CI) matrices, that is, the number of electronic states taken into account for each spin multiplicity. The former assessment is quite trivial: the appropriate active space will include all the Ln^3+^*n* 4f electrons distributed among the seven 4f orbitals; that is, a CAS(6,7) calculation needs to be performed in the present case regarding Eu^3+^. As far as the latter point is concerned, this is usually not discussed in detail in the literature,^61−63^ and even if so, it is done in a rather heuristic fashion.^17,19^

Differently from the TD-DFT black-box approach, the setup of a multireference numerical experiment is not at all a matter of routine. In fact, both the active space choice and the selection of the CI matrices dimensions imply, *a priori*, a rather deep understanding of the electronic properties of the investigated system. The Eu^3+^ 4f^6^ electronic configuration implies 3003 possible microstates, that is, ways of distributing six electrons in 14 spin–orbitals. This nominal degeneracy is lifted by the electron repulsion, SOC, and the crystal field in order of decreasing intensity. In the Russell–Saunders coupling scheme,^64^ the electron repulsion generates the ^2*S*+1^*L*(τ) terms with *S* and *L* corresponding to the total spin angular momentum and total orbital angular momentum quantum numbers, respectively (τ is an additional identifier discriminating between states with the same *S* and *L* quantum numbers). According to Hund’s rules,^65^ the free-ion ground state term for Eu^3+^ is the ^7^F_0_. The crystal field eventually present further reduce the 2*J* + 1 degeneracy of the ^2*S*+1^*L*(τ)*_J_* states according to the symmetry of the Ln^3+^ chemical environment.

A RASSI-CAS(6,7) calculation featuring a CI matrix of dimension 7 × 7 for electronic states with a spin multiplicity of 7 should describe appropriately the ^7^F*_J_* terms of the GS manifold. Moreover, the dynamic correlation inclusion (at the CASPT2 level) is unessential because we are not focusing on the ^5^D*_J_* excited states energies. In Table 1, the energies for the ^7^F*_J_* states calculated including seven septets as well as five quintets for tracking the ^5^D term are reported. Each ^7^F*_J_* free-ion state is split in 2*J* + 1 crystal field levels in the complex due to its low symmetry (*C*_1_); therefore, its energy has been taken as the barycenter of the manifold of levels within the same energy range. This is probably the most appropriate way to treat the electronic GS term, not only for the better agreement between theory and experiments but also for the lack of ambiguity compared to the TD-DFT calculations. The number and character of the output states are directly assignable to the expected theoretical levels. The comparison of CASSCF and CASPT2 results reveals minor differences for the ^7^F*_J_* states while the opposite is true for the ^5^D_0_ state where, as expected, dynamic correlation plays a relevant role. Indeed, in the CASPT2 framework, the reference state (i.e., the CASSCF wave function) directly interacts only with states differing by a single or double excitation.^18^ In a septet state only a limited number of single excitations preserve *S* = 3; at variance to that the CASSCF wave function may interact with a definitely larger number of states when a quintet is involved. In Figure 4 is reported the comparison between the experimental energy of the different ^7^F*_J_* manifolds (Figure 4, dotted lines) and the corresponding CAS(6,7)PT2 ones (Figure 4, solid lines). All these values are in Table 1. The inspection of Figure 4 testified the good agreement between experimental and CAS(6,7)PT2 values, especially low *J* values. This is consistent with results from Ungur and Chibotaru,^66^ who found that the appropriate description of the Er^3+^ complex ground state manifold actually requires the inclusion of second-order perturbations, and the CASPT2 results are significantly different from the CASSCF ones. These outcomes cannot be translated directly to our Eu^3+^ system because the ground state of Er^3+^ (4f^11^ configuration) is represented by a quartet term (^4^I), for which the number of possible single and double excitations is much larger than for our septet ground state.

**Figure 6:**
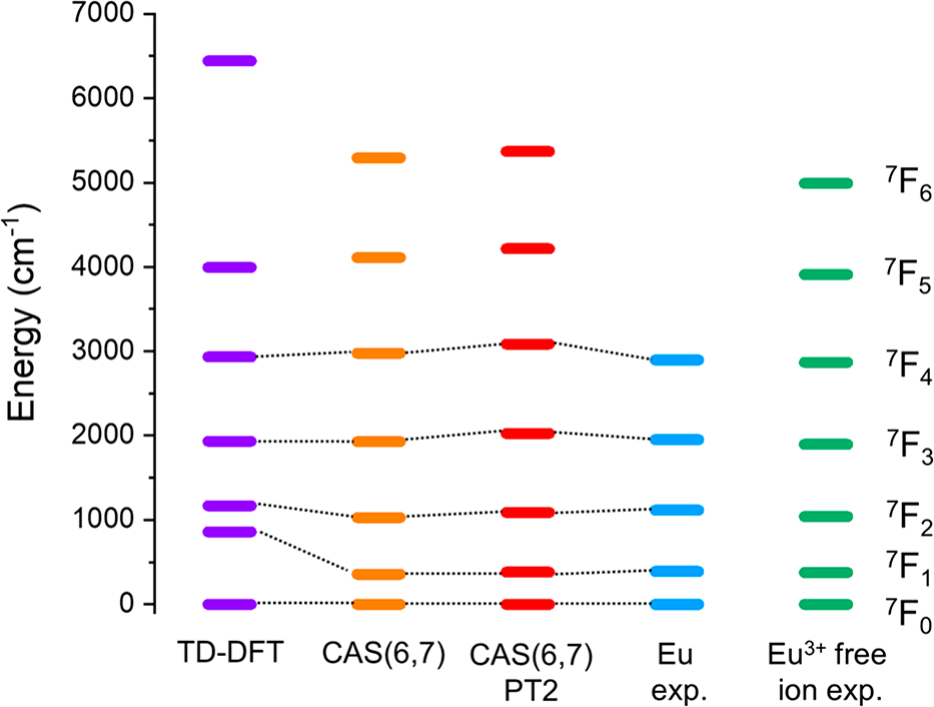
TD-DFT (violet bars), CAS(6,7) (orange bars), and CAS(6,7)PT2 (red bars) ^7^F*_J_* calculated energies for the Eu^3+^ model complex (see Table 1 for details). Eu^3+^ free ion experimental values (green bars) are also included for comparison with the Eu complex (blue bars).

The comparison between the diverse methods herein considered is schematically represented in Figure 6. Despite a slight overestimation of the energy of ^7^F*_J_* states with increasing *J*, multireference calculations provide satisfactory results. As far as the TD-DFT approach is concerned, the numerical agreement between experiment and theory is better for certain *J* values but worse for others. Once more, we emphasize that these TD-DFT calculations do not include spin–orbit effects, which are the leading term of interaction for these electronic states after electron repulsion. Finally, to definitively test the importance of the Eu^3+^ coordination sphere geometry, CASSCF and CASPT2 calculations are also performed on the Eu^3+^ isolated ion (see Table S2). In this case, there is a poor agreement between calculated values and the experimental ones obtained from the Eu^3+^ dopant in crystalline host matrices.^5,67^ The disagreement between experimental values and CASPT2 calculations is probability due to the fact that the energy terms acquired from data in crystalline matrices cannot be fully considered as “isolated ion” terms. Indeed, the effects of the surrounding chemical environment are indirectly included in the determination of the term energies. On the contrary, the CASPT2 calculations are performed on a truly isolated ion (Table S2), and the influence of the surrounding environment can be directly evidenced by comparison between CASPT2 outcomes on the Eu^3+^ isolated ion (Table S2) and on a molecular complex (Table 1).

When Eu^3+^-based luminescence is considered, transitions between the lowest-lying excited state (^5^D_0_) and the ground state manifold (^7^F*_J_*) are the most relevant. Therefore, it might be tempting to limit the states considered in the multireference calculation to the ^7^F*_J_* seven septets and the ^5^D*_J_* five quintets. This would be simply wrong because SOC allows the mixing of states with different (same) *L* and *S* (*J*) values. For instance, the ^7^F_0_ GS wave function includes the following main contributions:^68^ 93.4% ^7^F_0_ + 3.5% ^5^D_0_(1) + 2.8% ^5^D_0_(3) + 0.12% ^3^P_0_(6). Similarly, the wave function of the ^5^D_0_ emitter state has contributions from other states with *J* = 0. The inclusion of all possible states with *J* = 0 able to mix with ^5^D_0_ would imply, besides the seven septets, the presence of 140 quintets, 588 triplets, and 490 singlets. This is not only unrealistic but also unnecessary; in fact, the interaction we are dealing with is related to second-order perturbation theory,^18^ and it is well-known that the closer in energy the interacting states are the larger their mixing will be. We then do expect, knowing the layout of the lowest lying electronic terms,^5^ that ^5^D_0_ will strongly mix with septet states, other quintet states, and eventually low-lying triplet/singlet states, while its mixing with the high energy triplet/singlet states should be negligible. To quantify the mixing between ^5^D_0_ and other states, a series of RASSI-CAS(6,7)PT2 calculations have been performed on the Eu complex (see Table 2).

**Table 2:** ^5^D_0_ State Energies Calculated at the RASSI-CAS(6,7)PT2 Level for the Eu^3+^ Model Complex^a^

no.	no. of states (2*S* + 1)	^5^D_0_/cm^–1^
States with Different Multiplicities		
1	7(7) + 5(5)	20214
2	7(7) + 5(5) + 3(3)	17810
3	7(7) + 5(5) + 3(3) + 1(1)	17794
States with Different Multiplicities and Different Number of States		
1	7(7) + 5(5)	20214
4	7(7) + 31(5)	20055
5	7(7) + 42(5)	20018
6	7(7) + 49(5)	19987
7	7(7) + 62(5)	19947
8	7(7) + 77(5)	19895
9	7(7) + 140(5)	19676
10	7(7) + 140(5) + 3(3)	17504
11	7(7) + 140(5) + 31(3)	17506
12	7(7) + 140(5) + 3(3) + 1(1)	17313
13	7(7) + 140(5) + 31(3) + 1(1)	17311
14	7(7) + 140(5) + 31(3) + 20(1)	17315

a The labels identifying the calculations are reported in the first column. In the second column, the number of states included for each spin (in parentheses) are reported. The experimental value for Eu complex ^5^D_0_ is 17302 cm^–1^.^5^

**Figure 7:**
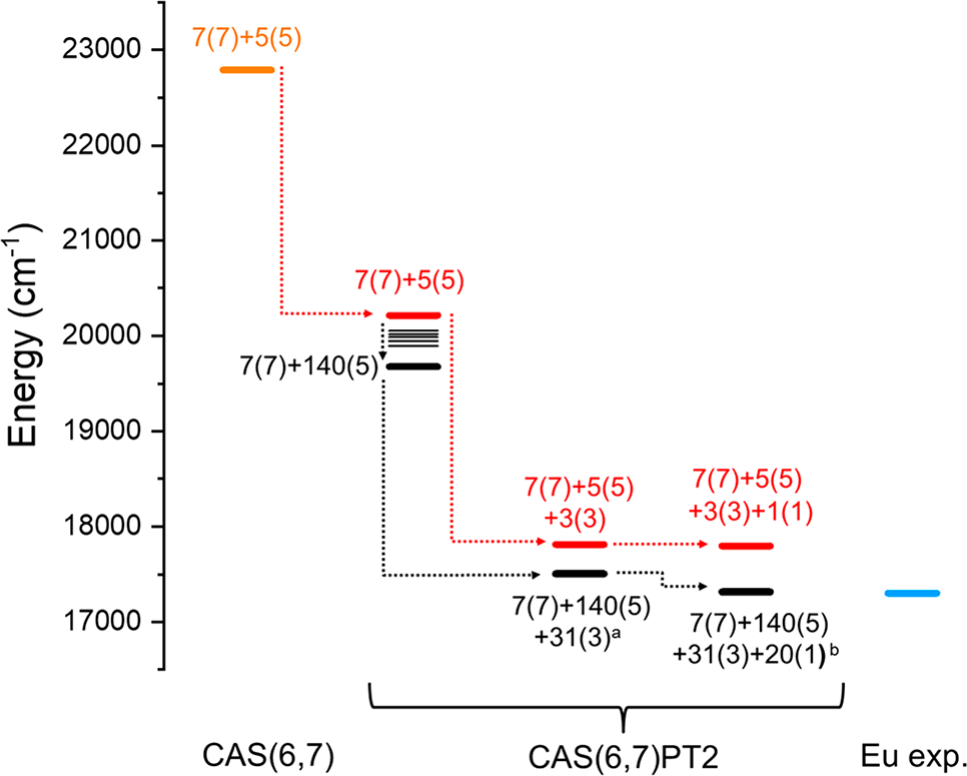
CAS(6,7) and CAS(6,7)PT2 ^5^D_0_ calculated energies for states with different multiplicities (red path) and for states with different multiplicities and a different number of states (black path). Calculations are performed on the Eu^3+^ model complex. The blue line is the experimental value for the Eu complex. ^*a*^Energies for 7(7) + 140(5) + 31(3) and 7(7) + 140(5) + 3(3) are equal (see Table 2, runs 10 and 11). ^*b*^Energies for 7(7) + 140(5) + 31(3) + 20(1) and 7(7) + 140(5) + 31(3) + 1(1) are very similar (see Table 2, runs 13 and 14) and are not distinguishable in the graph.

In the first set of calculations, the role of states with different multiplicities (quintets, triplets, and singlets) is considered (from run 1 to run 3 in Table 2). The base calculation (run 1 in Table 2) only features seven septets and five quintets, which is equivalent to taking into account the ground ^7^F and the excited ^5^D states. The ^5^D_0_ state is calculated at 20214 cm^–1^, definitively too high with respect to the experimental ^5^D_0_ energy, which is found at 17302 cm^–1^ for the Eu complex. Such a result ultimately testifies the poor description of the excited state. The mixing with other electronic terms with *J* = 0, for which Binnemans^5^ reports all the energies for levels below 40000 cm^–1^, seems to be a crucial factor. The lowest lying triplet state is ^3^P.^5^ Its inclusion in run 2 through the addition of three triplet states drops the ^5^D_0_ energy to 17810 cm^–1^, thus confirming the importance of this mixing. The addition of one singlet state (run 3) further improves the agreement, even if only marginally. States with different multiplicities contribute differently to the result. In particular, the inclusion of triplet states is more important than the singlet one. The reason is probably due to the higher energy of the singlet (above 40000 cm^–1^) that disadvantages, but not prevents, direct mixing with the ^5^D_0_ state. A graphical representation of the trend in these calculations can be found in Figure 7 (red path). An uncertainty of around 3 cm^–1^ has been found for these calculations by running them multiple times.

In the second set of calculations, in addition to states with different multiplicities, also the role of the number of states within the same spin multiplicity is investigated (from run 4 to run 14 in Table 2). The progressive addition of quintets up to the inclusion of all possible states with this multiplicity (140) significantly changes the ^5^D_0_ energy with an improvement of over 500 cm^–1^ (see Table 2, from run 4 to run 9). This trend is almost linear.^69^ As already demonstrated in the first set of calculations (run 2 in Table 2), the addition of triplets allows a better agreement with experimental value (a jump of around 2100 cm^–1^, run 10), but the inclusion of a larger number of triplets (the ^3^K (15) and ^3^I (13) terms, run 11 in Table 2) does not change significantly the ^5^D_0_ energy. This is likely due to the fact that the ^3^K_0_ and ^3^I_0_ levels are too high in energy (the lowest-lying levels for ^3^K and ^3^I are ^3^K_6_ (38780 cm^–1^) and ^3^I_6_ (38780 cm^–1^), respectively, while the terms with *J* = 0 are found well above 40000 cm^–1^),^5^ whereas the lowest lying ^3^P state (^3^P_0_, 32790 cm^–1^)^5^ is more easily accessible. These energy differences lead to a poor energy match with the ^5^D_0_ state for second-order perturbation mixing. Similarly to triplets, adding a singlet reduces the ^5^D_0_ energy by around 200 cm^–1^ (run 12 in Table 2).

This is an interesting difference with respect to the run 3, in which the addition of the singlet state did not produce an effect of this magnitude. However, the inclusion of a larger number of singlet states (run 14 in Table 2) does not change the energy of the ^5^D_0_ state in any meaningful way. We could suppose that the ^1^S_0_ state associated with the inclusion of this singlet does not mix directly with the ^5^D_0_ state but rather mixes with other states (other quintet states, ^3^P_0_), which in turn mix with the ^5^D_0_ state, contributing indirectly to the determination of its energy. Other high-energy triplets do not seem to mix with this singlet state significantly (runs 12 and 13). A graphical representation of the trend in these calculations can be found in Figure 7 (black path). Figure 7 clearly resumes from one side the role of the triplets, singlets, and quintets and from the other side the effects of a number of states involved in the ^5^D_0_ value calculations for the CAS(6,7)PT2.

Considering the data in Table 2 and Figure 7, it is possible to infer that: (i) the inclusion of the triplets (^3^P) strongly improves the agreement with the experimental value, as they mix directly with the ^5^D_0_ state; (ii) differently from the triplets, the addition of the singlet (^1^S) to the calculations with quintets and triplets only slightly affects the agreement with experimental value via an indirect mechanism; and (iii) the number of states is significant only for the quintets, while it is almost negligible for triplets and singlets, as only the lowest-lying term has an effect on the ^5^D_0_ state. A very good agreement between experimental and calculated values can be obtained considering all quintets and a minimal number of triplets (3) and singlet (1).

## 4 Conclusions

This study features advanced applications of *ab initio* quantum chemistry methods in the form of the nonroutine use of density functional theory based techniques as well as employment of multireference methods (CASSCF/CASPT2) for the rigorous treatment of the Eu^3+^ molecular complex. In particular, in the former point the absorption properties of the complex are studied; in the latter we address a number of inconsistencies in the literature regarding technical parameters in multireference calculations on Ln^3+^ ions, outlining the appropriate options on the base of theoretical arguments and calculated results.

The literature demonstrates the importance of second-order perturbation theory when considering excited states. Nevertheless, the role of the mixing and the choice of the relevant states are still under discussion. In this contribution, a general protocol based on a combination of DFT and multireference methods is presented to gain detailed information about Eu^3+^ electronic states. The shortcomings of DFT have been highlighted as well as some general guidelines for carrying out CASPT2 calculations. For the description of the GS manifold, static correlation is the leading term; therefore, a CASSCF calculation is enough, and CASPT2 is not necessary for the Eu^3+^ ion. When considering excited states, dynamic correlation in the form of second-order perturbation theory needs to be introduced. Because an electronic state can in principle mix with any other state with the same value of *J*, a series of benchmark calculations were performed to illustrate how significant this mixing is and to frame the appropriate way to carry out these calculations, since the literature is not in clear agreement on this point.

In particular, we have shown that for the ^5^D_0_ state energy calculation, two parameters are important: (i) the inclusion of states with different multiplicity and (ii) the number of states considered for each multiplicity. The relative weight of these parameters in improving the agreement with the experimental value is different. The inclusion of triplet and singlet states is crucial. The inclusion of a large number of states is necessary only for the quintets, while it is practically negligible for triplets and singlets.

To summarize, the finalized protocol for the determination of Eu^3+^-based emission properties in molecular complexes (the protocol evaluating ligand-based properties can be found in our previous study)^56^ consists of the following steps: (i) geometry optimization of the whole complex at the DFT/PBE level; (ii) evaluation of LMCT transitions at the TDDFT/SAOP level; and (iii) CAS(6,7)PT2 calculations on a model system which maintains the coordination sphere of the original complex, limited to 7 septet states without second-order perturbation effects for the ^7^F*_J_* ground state manifold and 7 septets, 140 quintets, 3 triplets, and 1 singlet for the accurate determination of the ^5^D_0_ emitter level.

The outcomes to this Eu^3+^ case study can be extended to other Ln^3+^ ions as well. As a rule of thumb, all states that can reasonably mix with the emitter level should be considered. In the absence of experimental data for the possible spectroscopic terms to be included in the CASPT2 calculation for the determination of the ^5^D_0_ state energy, a series of prescreening calculations on an isolated Ln^3+^ ion can be performed because its excited electronic levels are not expected to be greatly influenced by the presence of ligands. The appropriate configuration interaction (CI) matrices size can then be set from these preliminary calculations (Table S3).

## Supporting Information Available

Comparison between the ligand and the Eu^3+^ complex MOs in SAOP/TD-DFT transitions (Figure S1); most intense relevant SAOP/TD-DFT UV–vis electronic transitions for the Eu^3+^ complex (Table S1); ^7^F_*J*_ and ^5^D_0_ state energies calculated at the RASSI-CAS(6,7)PT2 level for the Eu^3+^ free ion (Table S2) and ^5^D_0_ state energies calculated at the RASSI-CAS(6,7)PT2 level for the Eu^3+^ free ion (Table S3) (PDF)
