# Supplementary material for: Multireference Ab Initio Investigation on Ground and Low-Lying Excited States: Systematic Evaluation of J–J Mixing in a Eu3+ Luminescent Complex
Source: Inorg Chem. 2020 Dec 15;60(1):315–24. doi: 10.1021/acs.inorgchem.0c02956 (PMC8769492; doi:10.1021/acs.inorgchem.0c02956)
Supplement: Supplementary file 1 — ic0c02956_si_001.pdf [file ic0c02956_si_001.pdf]

Multireference ab initio investigation on ground and low-lying excited states: systematic evaluation of J-J mixing in a Eu^{3+} luminescent complex.

Luca Babetto,[a] Silvia Carlotto,[a,b*] Alice Carlotto,[a] Marzio Rancan,[b] Gregorio Bottaro,[b] Lidia Armelao[a,b] and Maurizio Casarin[a,b*]

[a]Dipartimento di Scienze Chimiche, Università degli Studi di Padova, via F. Marzolo 1, 35131 Padova, Italy.

[b]Institute of Condensed Matter Chemistry and Technologies for Energy (ICMATE), National Research Council (CNR), c/o Department of Chemistry, University of Padova, via F. Marzolo 1, 35131 Padova, Italy

Correspondence: silvia.carlotto@unipd.it; maurizio.casarin@unipd.it

Figure S1. Comparison between the orbitals involved in the most intense SAOP/TD-DFT transition for the ligand (top) and the Eu complex (bottom). Even in the complex the transitions are still fully localized on the ligand, and the nature of the orbitals involved is analogue as that of the isolated form.

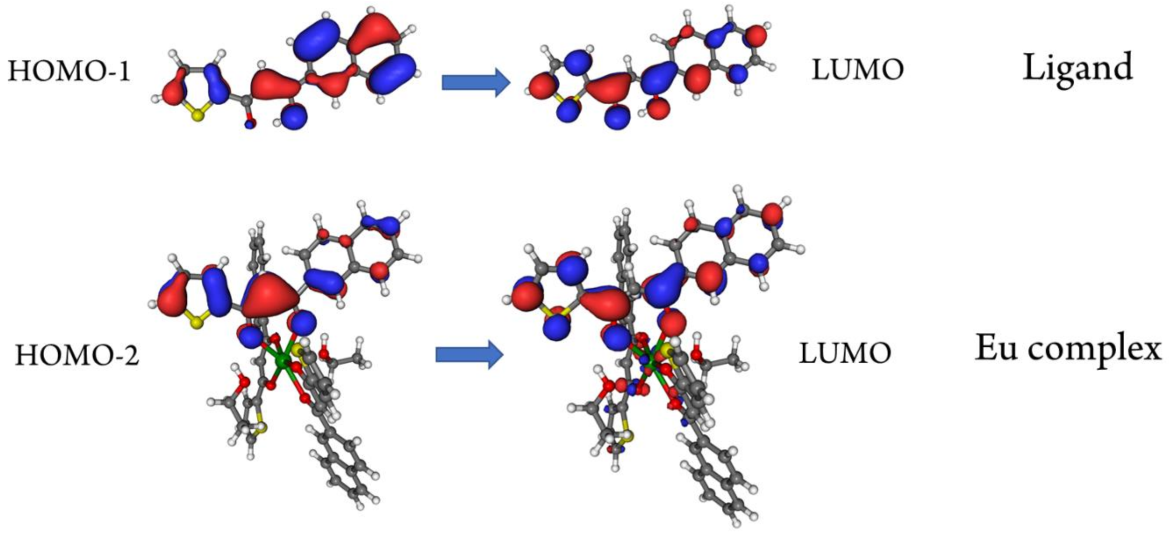

Table S1. Most relevant SAOP/TD-DFT UV-Vis electronic transitions for the Eu complex. Only transitions in the 300 – 480 nm range with normalized intensities higher than 20% of the most intense transition have been included.

wavelength (nm)	*normalised intensity*	$MO_i \longrightarrow MO_f$ (character)	MO_i	MO_f
418	0.30	HOMO ⟶ LUMO+6 (LMCT)	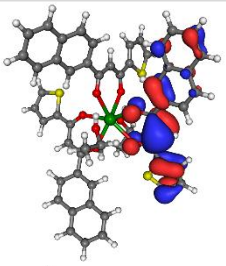	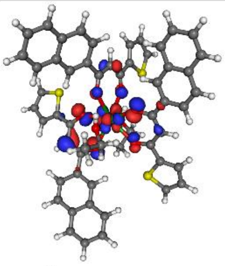
412	0.34	HOMO-1 ⟶ LUMO+6 (LMCT)	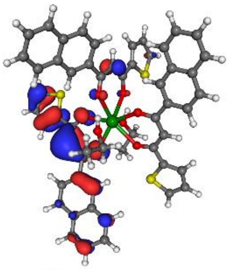	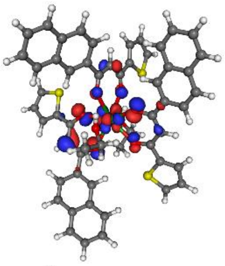
406	0.29	HOMO-2 ⟶ LUMO+6 (LMCT)	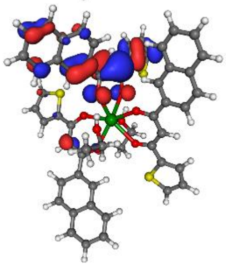	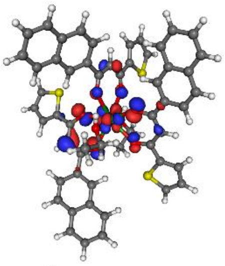
403	0.24	HOMO ⟶ LUMO+1 (L-centred)	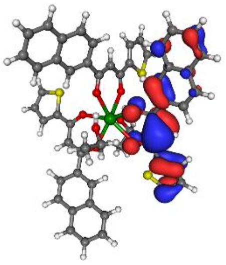	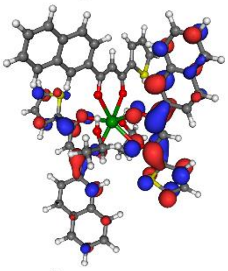
400	0.73	HOMO-1 ⟶ LUMO+2 (L-centred)	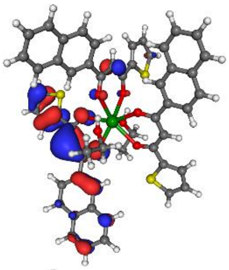	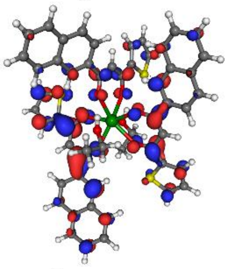
398	1	HOMO-2 ⟶ LUMO (L-centred)	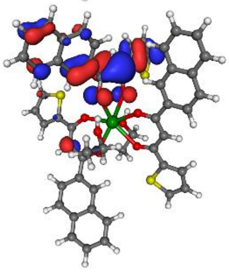	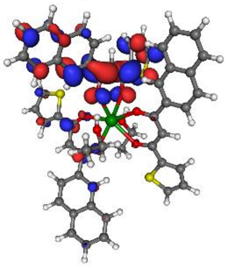

Table S2. ^7F_J and $^5D_0$ state energies (in cm^{-1}) calculated at the RASSI-CAS(6,7)PT2 level for the Eu^{3+} free ion. Each ^7F_J term for CAS calculations is taken as the barycentre of the respective manifold generated by crystal field splitting. Unfortunately, scalar relativistic TD-DFT/LB94 calculations systematically converge to fractional occupation numbers which are incompatible with the calculation of excitation energies in ADF. Forcing integer occupation numbers causes the SCF to not converge.

	CAS(6,7)	CAS(6,7)PT2	Eu^{3+} free ion exp.[5]
		Ground state	
$^7F_0$	0	0	0
$^7F_1$	382	406	379
$^7F_2$	1104	1168	1043
$^7F_3$	2099	2206	1896
$^7F_4$	3292	3431	2869
$^7F_5$	4625	4780	3912
$^7F_6$	6062	6219	4992
		Excited state	
$^5D_0$	22778	20441	17227

Table S3. $^5D_0$ state energies calculated at the RASSI-CAS(6,7)PT2 level for the Eu^{3+} free ion. The labels identifying the calculations are reported in the first column. In the second column, the number of states included for each spin (in parentheses) are reported. Experimental value for the $^5D_0$ state energy is 17227 cm^{-1}.[5]

#	number of states (2S+1)	$^5D_0$ / cm^{-1}
	States with different multiplicities	
1	7(7) + 5(5)	20441
2	7(7) + 5(5) + 3(3)	18265
3	7(7) + 5(5) + 3(3) + 1(1)	18086
	States with different multiplicities and different number of states	
1	7(7) + 5(5)	20441
4	7(7) + 31(5)	20383
5	7(7) + 42(5)	20354
6	7(7) + 49(5)	20325
7	7(7) + 62(5)	20296
8	7(7) + 77(5)	20253
9	7(7) + 140(5)	20081
10	7(7) + 140(5) + 3(3)	18019
11	7(7) + 140(5) + 31(3)	18018
12	7(7) + 140(5) + 3(3) + 1(1)	17850
13	7(7) + 140(5) + 31(3) + 1(1)	17852
14	7(7) + 140(5) + 31(3) + 20(1)	17852
